# A translational bioinformatic approach in identifying and validating an interaction between Vitamin A and CYP19A1

**DOI:** 10.1186/1471-2164-16-S7-S17

**Published:** 2015-06-11

**Authors:** Santosh Philips, Jing Zhou, Zhigao Li, Todd C Skaar, Lang Li

**Affiliations:** 1 School of Informatics and Computing, Indiana University-Purdue University Indianapolis, 46202, USA; 2 Harbin Medical University Cancer Hospital, Harbin, Heilongjiang, 150040, China; 3 Division of Clinical Pharmacology, Indiana University School of Medicine, Indianapolis, 46202, USA; 4 Center for Computational Biology and Bioinformatics, Indiana University School of Medicine, Indianapolis, 46202, USA

## Abstract

**Introduction:**

One major challenge in personalized medicine research is to identify the environmental factors that can alter drug response, and to investigate their molecular mechanisms. These environmental factors include co-medications, food, and nutrition or dietary supplements. The increasing use of dietary supplements and their potential interactions with cytochrome P450 (CYP450) enzymes is a highly significant personalized medicine research domain, because most of the drugs on the market are metabolized through CYP450 enzymes.

**Methods:**

Initial bioinformatics analysis revealed a number of regulators of CYP450 enzymes from a human liver bank gene expression quantitative loci data set. Then, a compound-gene network was constructed from the curated literature data. This network consisted of compounds that interact with either CYPs and/or their regulators that influence either their gene expression or activity. We further evaluated this finding in three different cell lines: JEG3, HeLa, and LNCaP cells.

**Results:**

From a total of 868 interactions we were able to identify an interesting interaction between retinoic acid (i.e. Vitamin A) and the aromatase gene (i.e. CYP19A1). Our experimental results showed that retinoic acid at physiological concentration significantly influenced CYP19A1 gene expressions.

**Conclusions:**

These results suggest that the presence of retinoic acid may alter the efficacy of agents used to suppress aromatase expression.

## Introduction

The Cytochrome P450 system consists of 57 enzymes, which are further classified into 18 families and 43 subfamilies based on sequence similarity[1]. They play a crucial role in the metabolism of various chemicals both endogenous and exogenous[2]. The members of the first three CYP families 1-3 are mainly involved in the metabolism of exogenous compounds such as medications, whereas the members of the other families are involved largely in the metabolism of endogenous compounds such as cholesterol, bile acids, steroid hormones and fatty acids. A given CYP enzyme can metabolize multiple substrates and a given substrate can be metabolized by multiple CYPs. Mutations or the absence of genes encoding the CYP enzymes can not only result in altered drug response but can also make an individual more susceptible to human disease such as glaucoma[3-7] and elevated cholesterol[8].Even a single mutation has the potential to alter the structure of these enzymes, resulting in altered activity or substrate specificity[9]. Furthermore, the co-administration of multiple drugs can influence the enzymes involved in their metabolism either through induction or inhibition [10]. Age and sex as well can influence CYP activity, studies have shown that CYP3A4 activity is higher in adults compared to fetus [11] and that women metabolize CYP3A4 faster than men [12,13]. A wealth of information on the CYP variants is available at the Human Cytochrome P450 Allele Nomenclature website [14]. Despite the presence of this large amount of information it is still challenging to optimize therapy to meet an individual's needs, especially with the increasing usage of supplements and herbal medications.

One of the challenges of personalized medicine is to identify or fine tune drug combinations without drastically affecting the metabolic pathway of either. There are numerous studies that have shown that the activity of these enzymes are influenced by various upstream regulatory mechanisms [13,15-24], which in turn can potentially influence drug response. Despite the use of various patient characteristics there still exist a substantial amount of variations in drug response and mainly due to the nature and combinations of sources of variation. One such factor is the increasing use of dietary supplements that are not always taken into account while drugs are prescribed, which can potentially alter Cytochrome P450 activity [25,26]. The mining of previously published literature across various disciplines has been very useful and effective in identifying potential drug interaction and rofecoxib is an excellent example, where the drugs toxic effect was present in literature before the drug was recalled [27]. Thus translational bioinformatics methods summarizing the literature data have proven to be an effective way of uncovering interactions that could be beneficial or harmful. In our study we were able to identify a correlation between retinoic acid and aromatase gene expression through bioinformatics analyses of existing databases. We were able to functionally validate this bioinformatic prediction in three different cell lines using physiological concentrations of retinoic acid. Our studies show that retinoic acid substantially alters the expression of the aromatase gene.

## Methods

### CYPs and their super-regulators

For this study we choose seven CYP subfamilies (CYP1A, CYP2A, CYP2B, CYP2C, CYP2D, CYP2E, and CYP3A) that are mainly responsible for metabolizing more than 90% of drugs as well as CYP19A1 that is largely involved in the biosynthesis of estrogens. In order to identify compounds that indirectly affected the CYP activity/expression we used previously published endogenous CYP regulators[13,15-24] from the literature and those that had a significant influence over the expression as well as activity of these CYP enzymes from the human liver bank gene expression quantitative loci data set[28]. These endogenous CYP regulators that had a direct effect on CYP enzymes were used as seed to identify compounds (Super-Regulators) that in turn influenced their regulation.

### Identification of compounds that influence CYP regulators

The endogenous CYP regulators were uploaded into Metacore (Thomson Reuters, NY, USA), a web based computational tool backed by text mining capabilities to build a highly interconnected network of CYP regulators and compounds that influenced their expression. Each node in the network represented a CYP regulator or a compound and the edges represented the interaction between the two denoting either an activation or inhibition. Not all CYP regulators were associated with upstream compounds. The CYP regulators that were not associated with any compounds and compounds that had fewer than 3 edges were eliminated from further analysis. The CYP regulators from the above list (compounds (edges =>3) - CYP regulator) were used to build a second network along with the 10 CYP enzymes, namely CYP1A2, CYP2A6, CYP2B6, CYP2C19, CYP2C8, CYP2C9, CYP2D6, CYP2E1,CYP3A4, AND CYP19A1 to further confirm the interactions between them. The results from the above two networks namely the [Compounds (edges =>3) - CYP regulator] and [CYP regulator- CYP enzyme] was merged to form the final network using Cytoscape[29] to represent the overall interaction between the compounds, CYP regulators and CYP enzymes. Using this network the path from a given compound to its terminal leaf, which was either a CYP regulator or CYP enzyme was traced, thus predicting the interaction between the compound and CYPs.

## Influence of retinoic acid on DAX1/CYP19A1 gene expression

### Cell culture and treatment

Three cell lines namely, JEG3 (Placental Cancer), HeLa (Cervical Cancer), and LNCAP (Prostate Cancer) were chosen to study the expression of the CYP19A1 (Aromatase) and DAX1 genes. The cells were plated in six T25 flasks at a density of 0.25 × 10^6 ^cells/ flask and grown in DMEM with 10% FBS. After 24 hours the media was removed and the cells were washed 3 times with DMEM containing 10% charcoal stripped FBS and cells were then allowed to grow in the new media. The media was replaced with fresh media every 24 hours for two more days. After 72 hours of initial media change the cells in each of the six flasks were treated with either vehicle (0.01% Ethanol) or All Trans Retinoic Acid (ATRA) (Sigma-Aldrich, USA) at 0.1nM. 1nM, 10nM, 100nM and 1000nM respectively.

### RNA extraction, cDNA synthesis and gene expression

After 24 hours of treatment the cells were harvested and RNA extracted using miRNeasy Kit (Qiagen Inc., USA) according to the manufactures protocol. The RNA was then quantified using Quant-IT Kit (Life Technologies, USA) on the Qubit Fluorometer (Life Technologies, USA) according to the manufactures protocol. The cDNA was synthesized using the QuantiTect Reverse Transcription Kit (Qiagen Inc. USA) according to the manufactures protocol from 1ug of RNA. The gene expression for CYP19A1 and DAX1 was measured with the respective Taqman Gene Expression Assays (Life Technologies, USA) on the iCycler instrument (Bio-Rad Inc., USA) in accordance with the manufactures protocol.

## Results

### CYPs and their regulatory network

**Figure 1:**
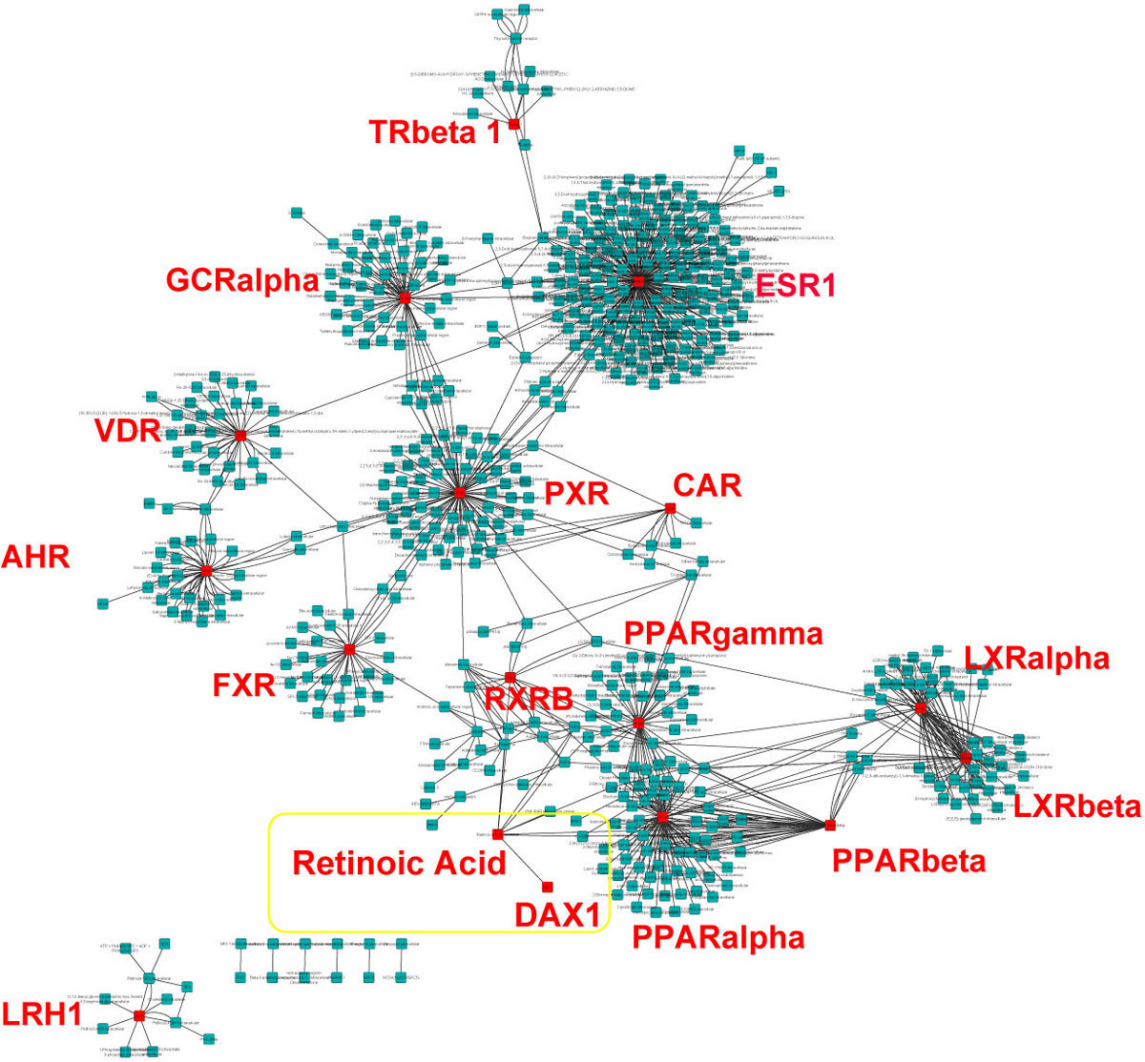
**Compound - CYP Regulator Network**. The above figure depicts the overall network consisting of 868 interaction between the various compounds and CYP regulators that they affect. The density of the cluster is proportional to the number of compounds that influence its activity, and the nodes between clusters represents the compounds that influence more than one CYP regulator. The top 15 clusters were formed around ESR1, PXR, PPARalpha, LXRalpha, GCRalpha, LXRbeta, AHR, PPARgamma, PPARbeta, VDR, FXR, RXRbeta, LHR, CAR and TRbeta (the respective nodes are highlighted in red and interaction between retinoic acid and DAX1 is highlighted by the yellow rectangle).

**Figure 2:**
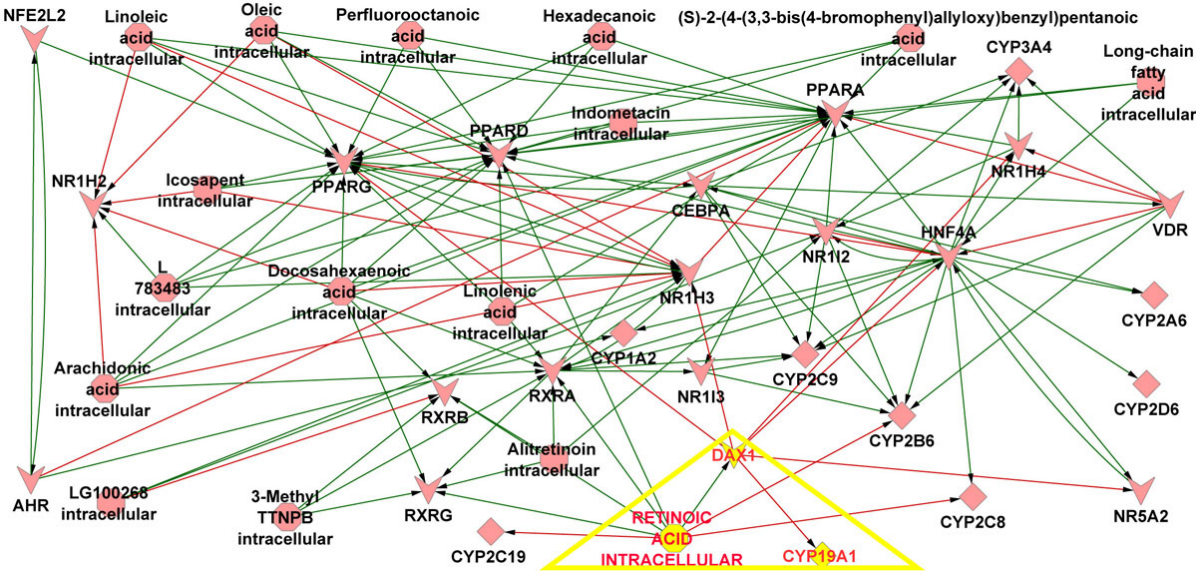
**Compound - CYP Regulator - CYP Network**. The above network shows the interaction between the Compounds, CYP regulators and CYPs. Green arrows represent up regulation and red arrows represent down regulation of the respective gene. The interaction between retinoic acid, DAX1 and CYP19A1 is highlighted by the yellow triangle.

The initial network between the compounds and CYP regulators consisted of 868 edges between the compounds and CYP regulators, with 15 large clusters (Figure 1). The top 15 clusters were around the following CYP regulators namely, ESR1, PXR, PPARalpha, LXRalpha, GCRalpha, LXRbeta, AHR, PPARgamma, PPARbeta, VDR, FXR, RXRbeta, LHR, CAR and TRbeta. The number of compounds that formed these cluster ranged from 187 to 7, thus representing the extent to which a single CYP regulator can be influenced by multiple compounds. This network was further reduced by eliminating compounds that had less than 3 edges with other CYP regulators. The final regulatory network mainly consisted of the 134 edges between 42 nodes which included 9 CYPs, 16 CYP regulators and 17 compounds (Figure 2). All of the drug metabolizing CYP enzymes including the CYP19A1 had either a compound or CYP regulator upstream. Only CYP2E1 did not have any compound or CYP regulators associated with it. The resultant network clearly indicated the potential influence of retinoic acid (Vitamin A) on the expression of CYP19A1 (Aromatase) through DAX1.

### Influence of ATRA on CYP19A1 (Aromatase) and DAX1 genes in the various cell lines

**Figure 3:**
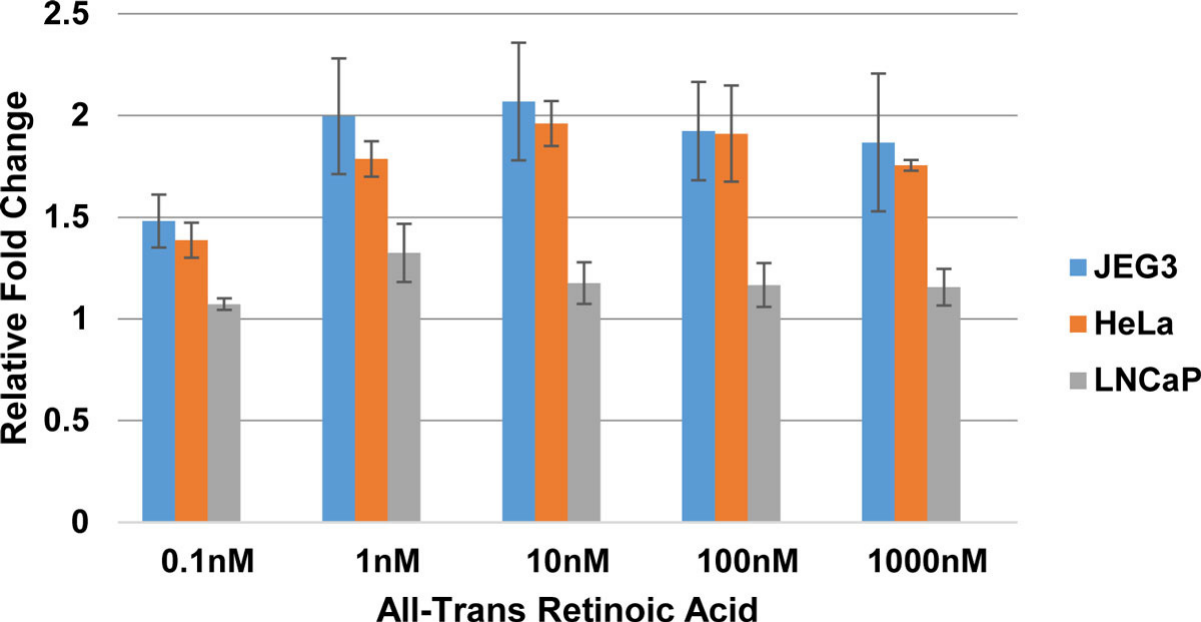
**Relative fold change of Aromatase gene in response to various concentrations of ATRA**.

**Figure 4:**
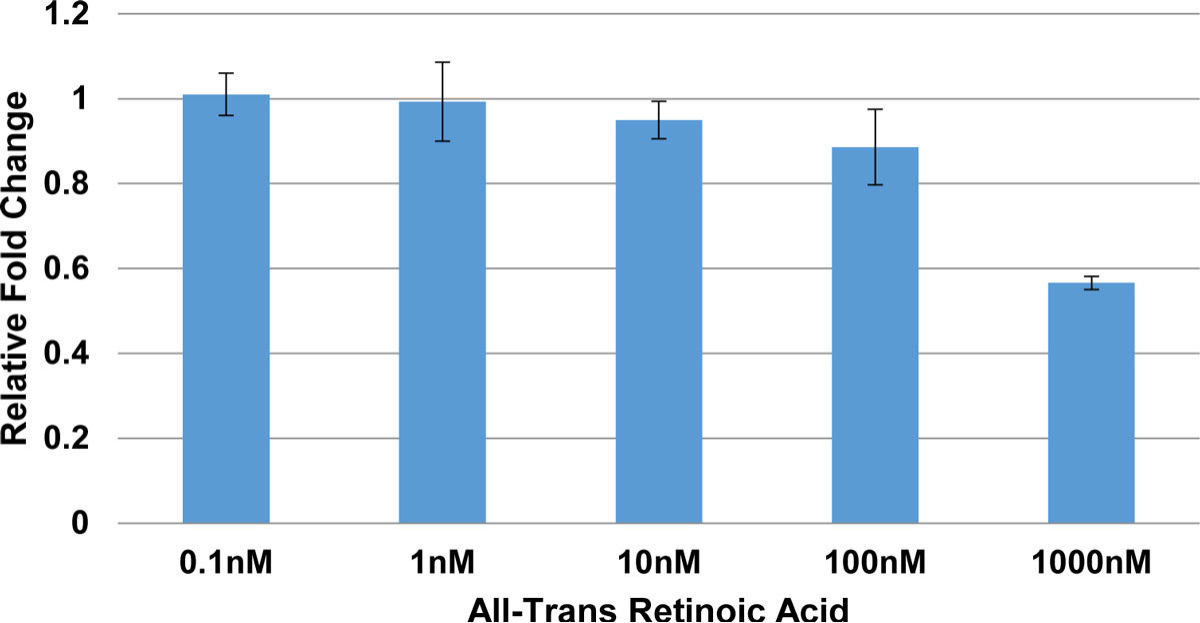
**Relative fold change of DAX1 gene in response to various concentrations of ATRA**.

The above hypothesis that retinoic acid alters the expression of aromatase gene was experimentally verified using three different cells lines, namely JEG3, HeLa and LNCaP. Each cell line was treated with various concentrations of ATRA ranging from 0.1nM to 1uM, which included the physiological concentration at which retinoic acid is found in humans. The physiological concentration of retinoic acid in human plasma is around 4.9 ng/ml and all-trans retinoic acid (ATRA) accounts for ~75% of the total[30]. After a 24 hour treatment period the cells were harvested, RNA extracted and the expression was measured for CYP19A1 and DAX1 using the respective Taqman gene expression assays. The expression of aromatase gene increased proportionally with increasing concentrations of ATRA and tapered off at 10nM ATRA (Table 1, Figure 3). DAX1 expression was observed only in the HeLa cell line showing a decrease in activity with increasing concentration of ATRA (Figure 4). The above experiments were performed in triplicates on different days for each cell line.

**Table 1:** F-statistic along with the p-value for the effect of ATRA on the expression of CYP19A1 (aromatase) and DAX1 across the each cell lines.

Cell Line	Gene	*F*(5/12)	*p-value*
**HeLa**	**CYP19A1**	9.7750	0.0007
	
	**DAX1**	8.1982	0.0014

**JEG3**	**CYP19A1**	2.8328	0.0647

**LNCaP**	**CYP19A1**	1.4003	0.2920

A one-way between treatments ANOVA was conducted to compare the effect of retinoic acid on the expression of CYP19A1 and DAX1 in the three different cell lines. There was a significant effect of retinoic acid on the expression of CYP19A1 and DAX1 at the P < 0.05 level in the HeLa cell line (Table 1). Further, post hoc comparison using the Tukey test showed that the fold change for treatments (0.1nM, 1nM, 10nM and 100nM) were significantly different from treatment at 1000nM. Thus indicating that the expression of aromatase gene proportionally increased with an increasing concentration of retinoic acid reaching 100nM, which included the physiological concentration at which retinoic acid is present in the human body.

## Discussion

In the current study we were able to identify 868 interactions between various chemical compounds and cytochrome P450 regulators. We choose to follow the interaction of retinoic acid on aromatase enzyme because of its possible significant application towards personalized medicine in endocrine therapy. The cell lines chosen for this study are known to express CYP19A1 and DAX1(HeLa). In the cell experiments, we found that retinoic acid up-regulates the aromatase enzyme. Retinoic acid a metabolite of Vitamin A is very commonly found in various foods and dietary supplements. Aromatase is a key enzyme involved in the biosynthesis of estrogens [31,32], which can catalyze the progression of estrogen-dependent breast cancers. The levels of aromatase activity and mRNA expression are higher in the breast cancer tissue than in normal tissue [33-35]. In addition to the ovarian supply of estrogens, aromatase enzyme is also involved in the local production of estrogens through the conversion of circulating adrenal androgens [36], thus having an immense potential to fuel estrogen receptor positive breast cancer. DAX 1(dosage-sensitive sex reversal adrenal hypoplasia congenital critical region on the X-chromosome gene 1) is an orphan member of the nuclear receptor family [37,38], and functions as global anti-steroid factor and represses the expression of many enzymes involved in the steroidogenic pathway, including aromatase [39,40]. The expression of DAX 1 has been reported in breast cancers [41,42], although it's exact mechanism is not fully understood. Aromatase inhibitors were developed and widely utilized to treat endocrine tumors[43], especially breast cancer with estrogen receptor positive patients. Therefore, the up regulation of aromatase would in turn result in higher levels of estrogens, and could possibly stimulate the endocrine tumor growth. Most importantly, the usage of Vitamin A could reduce the effectiveness of aromatase inhibitor treatment for cancer. Given the fact that Vitamin A is so commonly found in various food/supplement source, the chance for its potential influence is higher, thus we chose to validate its influence on aromatase expression.

Our initial bioinformatics finding using the MetaCore database revealed that the presence of retinoic acid caused an up regulation of DAX1 and which in turn caused the down regulation of aromatase. In our functional study, only one cell line, namely the HeLa cells showed expression for DAX1. HeLa cells treated with retinoic acid showed a down regulation of DAX1 and an up regulation of aromatase expression. Though not in the exact same direction as the bioinformatics prediction, the overall experimental outcome in HeLa cells does bring out the possibility that when present, DAX1 expression is inversely related to CYP19A1 expression in the presence of retinoic acid. The other two cell lines did not show any expression for DAX1 but did show that retinoic acid up regulated CYP19A1 expression. DAX1 is an orphan member of the nuclear receptor superfamily of transcription factors, whose disruption has been linked with increase expression of aromatase enzyme [39,44]. Even though the cell lines chosen for this study are from extra gonadal sites it clearly shows that retinoic acid has a significant influence on aromatase expression in the presence or absence of DAX1. This finding is of importance as the use of aromatase inhibitors in treating breast cancer is widespread [45,46]. Though aromatase inhibitors are a gold standard in treating ER-positive breast cancer, resistance to this therapy still requires the use of other modes of suppression of intra-tumoral estrogen production[36], thus calling for further investigation into the underlying cause of resistance.

## Conclusions

In this paper, we have shown that the use of curated literature data is valuable in discovering novel drug enzyme interactions, and potential clinically significant drug interactions. Our primary contribution is the established feasibility of this translational bioinformatics approach in detecting novel drug interaction signals.

## Competing interests

The authors declare that they have no competing interests.

## Authors' contributions

LL and ZL guided the study. SP performed the bioinformatics and data analysis, designed and carried out the wet lab experiments and wrote the manuscript. TS provided the wet lab support. All authors read and approved the final manuscript.

## Declarations

Publication charges for this article have been funded by Dr. Lang Li.

This article has been published as part of *BMC Genomics *Volume 16 Supplement 7, 2015: Selected articles from The International Conference on Intelligent Biology and Medicine (ICIBM) 2014: Genomics. The full contents of the supplement are available online at http://www.biomedcentral.com/bmcgenomics/supplements/16/S7.
